# Accuracy of Dysphagia Standard Assessment (DSA®) bedside screening test: a flowchart for patient eligibility

**DOI:** 10.1007/s10072-022-06175-9

**Published:** 2022-06-04

**Authors:** Laura Mariani, Armando Cilfone, Maria Nicastri, Lucia Libera Pipitone, Federica Stella, Marco de Vincentiis, Antonio Greco, Patrizia Mancini, Lucia Longo, Giovanni Ruoppolo

**Affiliations:** 1 Department of Sense Organs, Otorhinolaryngology Section, Sapienza University of Rome, Policlinico Umberto I, Viale dell’Università, 33, 00161 Rome, Italy; 2 I.R.C.C.S. San Raffaele Pisana, Rome, Italy

**Keywords:** Oropharyngeal dysphagia, Screening, FEES, Deglutition disorders, Nutrition, Swallowing

## Abstract

**Background:**

Oropharyngeal dysphagia (OD) screening tests have improved patient management; however, the complex applicability and high percentage of false negatives do not allow these tests to be considered completely reliable if not supported by an instrumental investigation. The aim of the present study is to evaluate an OD screening test, the Dysphagia Standard Assessment (DSA®) with different volumes and viscosities.

**Materials and methods:**

Prospective study of 72 patients evaluated for suspected OD through a double-blind methodology conducted by two operators. All patients underwent fiberoptic endoscopic evaluation of swallowing (FEES) as a reference test and a separate DSA® test. DSA® was performed by administering boluses with different viscosities, with the signal of interruption of the test being: onset of the cough reflex, wet voice after swallowing, and/or desaturation of O2 ≥ 5%. The Penetration-Aspiration Scale (PAS) was evaluated by FEES. The cut-off identified to diagnose OD was PAS ≥ 3.

**Results:**

The test showed an accuracy of 82%, a sensitivity of 0.93 (95% C.I. 0.84–0.97), and a specificity of 0.78 (95% C.I. 0.67–0.87); positive predictive value 0.55 (95% C.I. 0.43–0.67); negative predictive value 0.97 (95% C.I. 0.90–0.99), positive likelihood ratio 4.37 (95% C.I. 3.6–5.2); likelihood negative ratio 0.08 (95% C.I. 0.06–0.09).

**Conclusions:**

According to the preliminary results, the test showed good outcomes in determining the presence or absence of OD with a wide spectrum of applicability with some limitations that could be overcome by the selection of a target population. For this reason, a flowchart to address patient eligibility was developed.

## Introduction

​​Oropharyngeal dysphagia (OD) is a widespread issue that affects more than 40 million people in Europe and more than 16 million in the USA with various pathological conditions and the percentage of affected patients increasing [1].

Early diagnosis and treatment of OD avoid major complications such as malnutrition and dehydration, social isolation, and aspiration pneumonia, which is the most common cause of death among individuals with dementia [2–4], and also improves cost-effectiveness as a result of the reduction in the length of hospital stay [5]. It is therefore important not only to diagnose but also to prevent the development of this condition, thereby avoiding misdiagnosis and treatment failure [1, 4]. Currently, in clinical practice, patients with suspected aspiration are identified by screening tests and/or by instrumental evaluation using the video fluoroscopic study of swallowing (VFSS) or by fiberoptic endoscopic examination of swallowing (FEES) [6]. Several bedside screening tests (BST) have been designed to assess swallowing and can be performed by a speech therapist (ST) and/or by trained healthcare professionals using water and food of different consistencies and viscosities. BST represent a low-cost approach that does not require specialized equipment, have demonstrated reasonable sensitivity, are quick to perform, safe, simple, and easily repeatable, but the reproducibility and consistency of these protocols have not been established and have been shown to be poor at detecting silent aspiration [7].

On the other hand, FEES and VFSS can be performed in the most at-risk cases. VFSS is a dynamic radiological investigation performed using video recording that allows specialists to evaluate the act of swallowing and the esophageal morphology and motility during the administration of small boluses of liquid and/or solid baryta meal, thereby providing anatomical and functional information [8]. FEES is performed with the aid of a fiberoptic endoscope inspecting the oral cavity, orofacial praxis, bite strength, salivation management, neck musculature, and laryngeal elevation. A flexible fiberscope is introduced through the nasal fossa, and once the hypopharynx is reached, a series of investigations are performed including the presence of mucosal residue, lingual strength and propulsion, the different phases of swallowing through the ingestion of semi-solid and liquid substances, and the reflexes of cough and laryngeal adduction are evaluated [9]. Nevertheless, both methods require expensive resources, have a significant investment of time and planning, and need skilled operators and specialized equipment. Therefore, in an emergency scenario or first access to medical health care, dysphagia screening tests have provided the basic support for early diagnosis of dysphagia in patients with a high-risk of OD.

BST for early identification of dysphagia are highly recommended by stroke guidelines [10] and are suggested by the literature in neurodegenerative diseases [6]. The water swallowing test (WST) is habitually used for simplicity and speed of execution but variable results regarding the reliability of the test have been found in the literature. A meta-analysis [11] showed that in WST, sensitivity ranged from 27 to 85%, while specificity ranged from 63 to 88%. Combining WST with oxygen desaturation led to sensitivities between 73 and 98% and specificities between 63 and 76%. Alternatively in swallowing tests using different viscosities, sensitivity ranged from 41 to 100% and specificity from 57 to 82%.

Overall, several studies have shown that [12–16] swallowing tests using different viscosities seem to have a greater reliability and validity even if not exempted from a proportion of false positives and negatives. Although the causes of these negative responses to the tests have not been investigated, this has not lead to a consensus leading to a universally accepted protocol on the use of one or other test. The primary outcome of our study was to assess the reliability and validity of a swallowing test using different viscosities associated with the measurement of saturation in subgroups of patients with different diagnoses and suspected OD. The secondary outcome was to identify, where present, subgroups of patients with negative response to test, whose characteristics might shed light on the limits of the test’s sensitivity and specificity.

## Materials and methods

### Study design

The OD was investigated using a double-blind methodology between two operators. FEES was performed by two independent deglutologists. On the same day, a different viscosity screening test was performed at two separate times on all of the subjects, by one speech and language pathologist (SLP) blinded to the results of FEES. The FEES results were considered a reference test to establish the presence and severity of OD. In addition, the complete medical history for all patients was collected.

### Sample

The study population included twenty healthy volunteers to assess normal swallowing physiology and seventy-four patients with suspected OD, investigated by means of bedside evaluations between June 2018 and July 2019 in different wards of the Policlinico Umberto I Hospital in Rome. Our study included 25 patients who had cerebrovascular disease, 24 patients with neurodegenerative diseases, 11 patients with neurological diseases, and 12 with other diseases (Table 1). Exclusion criteria were patients with Glasgow Coma Scale (GCS) < 14, with psychiatric disorders, and who had head and neck cancer surgery. Two patients with previous glossectomy for oral cancer were also excluded. These 2 cases were excluded because, in accordance with recent evidence, it is believed that post-surgical oral dysphagia should always make use of instrumental evaluation as there is no international consensus on which screening for dysphagia is preferred in patients with head and neck cancer [17].

**Table 1:** Sample

	*N*
Cerebrovascular disease	25
Neurodegenerative disease	24
Amyothrophic lateral sclerosis	6
Multiple sclerosis	2
Senile dementia	6
Parkinson’s disease	10
Neurological disease	11
Myasthenia gravis	3
Myotonic dystrophy type 1	1
Oculopharyngeal myopathy	2
Spastic Tetraplegia	1
Facioscapulohumeral muscolar dystrophy	1
Infantile spastic cerebropathy	1
Inclusion body myositis	1
Idiopathic torsion dystonia	1
Other diseases	12
Neurosurgery	2
Post-intubation dysphagia for respiratory distress	2
Neoplastic cachexia	2
Multicentric Castleman disease	1
Mild traumatic brain injury	2
Meningioma	1
Partial gastrectomy	1
Gastroesophageal reflux disease	1
Total	72

### Index test

**Fig. 1:**
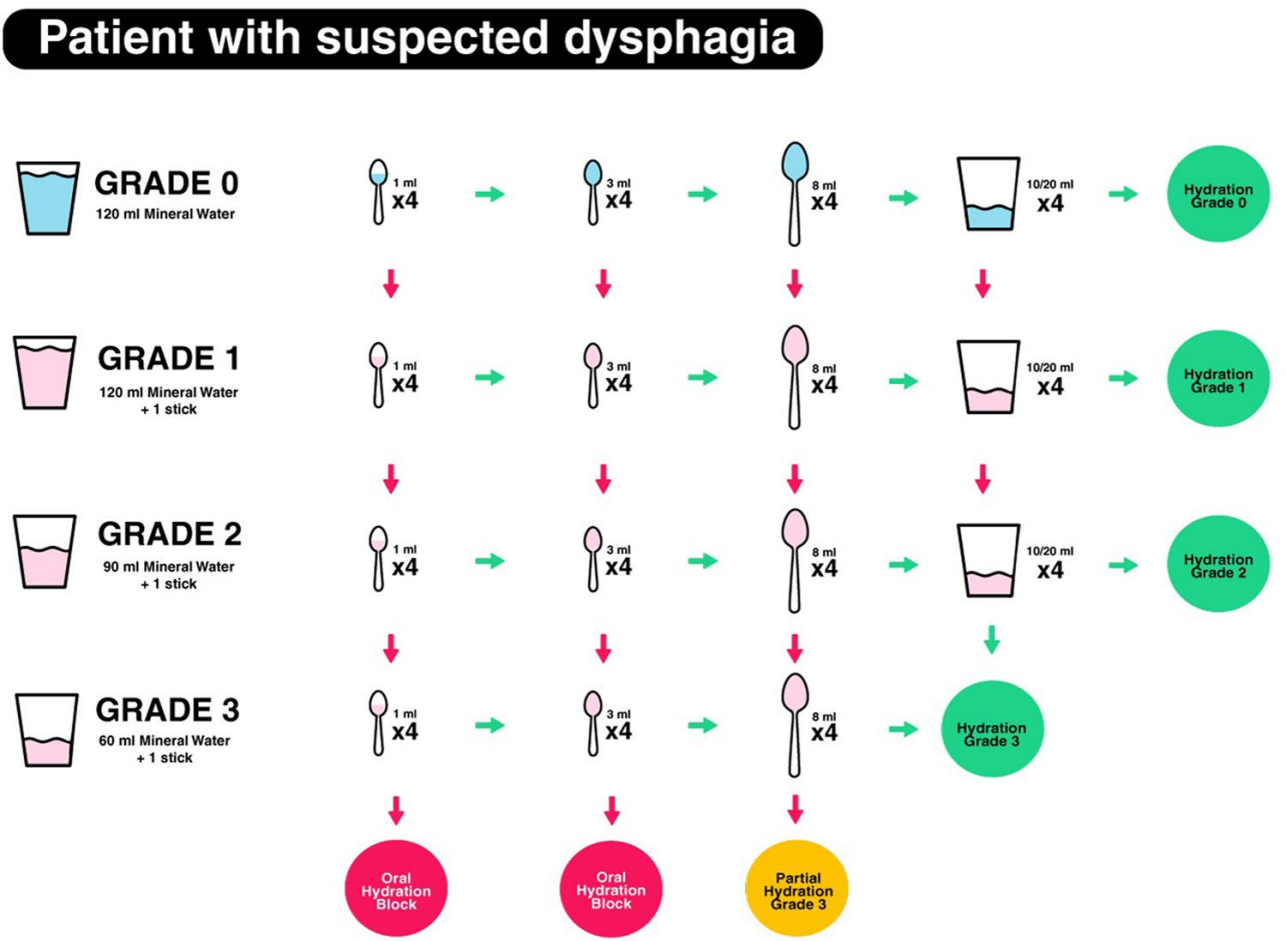
The screening test for patients with suspected dysphagia with 4 different liquid volumes and consistencies (grades) ranging from natural water (grade 0) to thickened or gelled water (grade 3)

All patients underwent a volume and viscosity screening test (DSA®—Dysphagia Standard Assessment; Nutrisens* medical) designed to protect the patient from aspiration by administering a bolus progressively increasing or decreasing in volume and viscosity. The purpose of the test was to identify the most suitable consistency for liquids based on the patient’s swallowing ability. The test included the following materials: three sachets of powder to make a gel with water, two spoons (3 ml and 8 ml), four glasses of natural cold water (2 glasses of 120 ml and 2 glasses of 90 ml and 60 ml, respectively), one 100-ml syringe to measure the water, a flexible straw to introduce liquids into the mouth where using a spoon was difficult or impossible, and finally one pulse oximeter. The patient was seated or semi-seated with their head bent slightly forward. The screening test included 4 different liquid volumes and consistencies (grades) ranging from natural water (grade 0) to thickened or gelled water (grade 3) (see Fig. 1). Grade 0 corresponded to performing swallowing (120 ml of water), grade 1 to likely aspiration risks (120 ml of water and one sachet of powder), grade 2 to more significant and frequent aspirations (90 ml of water and one sachet of powder), and grade 3 to significant and frequent aspirations (60 ml of water and one sachet of powder). The procedure would start at grade 0 or grade 3. Starting at grade 0 was suitable for all patients who had difficulty swallowing, whereas patients who already had swallowing disorders started at grade 3.

Starting at grade 0, the patient had to swallow 4 times half of a 3-ml teaspoon of natural water. If the patient coughed at the second swallow of half a 3-ml teaspoon of water, the caregiver continued the test by administering 4 times half of a 3-ml teaspoon of grade 1 water. If there were no problems, they increased the volume of the intake by administering 4 times a whole teaspoon of grade 1. The test was continued with 4 times of an 8-ml spoonful. If the patient was coughing, they would pass to grade 2 by administering 4 times of an 8-ml spoonful. If the patient swallowed correctly, they had to take 4 sips from the glass and if there were no problems the recommendation was for grade 2 (Fig. 1).

**Fig. 2:**
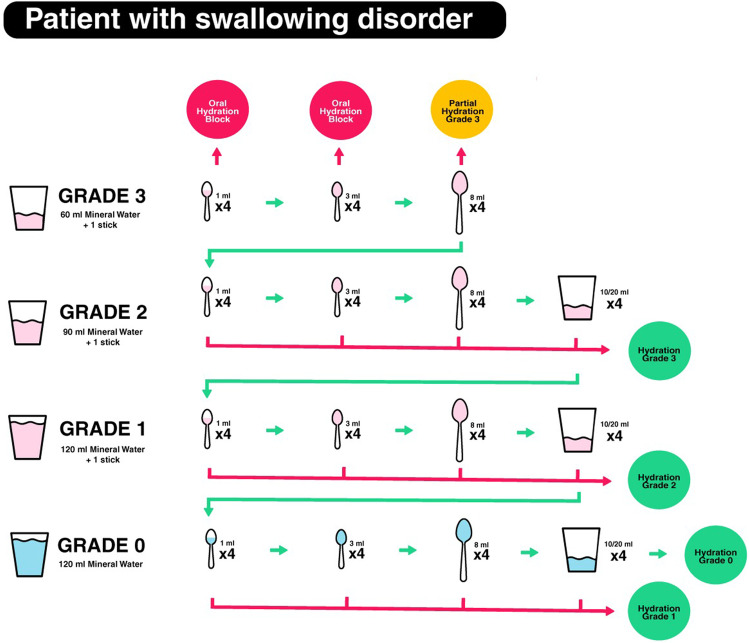
The screening test for patients with swallowing disorder with 4 different liquid volumes and consistencies (grades) ranging from natural water (grade 0) to thickened or gelled water (grade 3)

If starting at grade 3, the patient had to swallow 4 times half of a 3-ml teaspoon of thickened water with grade 3. If there were no problems, the test continued with the patient taking 4 times of an 8-ml spoon of grade 3. If completed without aspiration, they went to grade 2. If the subsequent swallowing did not give problems, they went to grade 1. If the patient coughed while drinking a spoonful, hydration with grade 2 liquids was recommended (Fig. 2).

During bolus administration, cough, wet voice after swallowing, and a desaturation of O2 ≥ 5% were considered significant enough for the risk of aspiration or penetration and were criteria for stopping the test.

### Reference test

All patients underwent a fiberoptic endoscopic evaluation of swallowing (FEES) by two experienced deglutologists using a flexible fiberscope (EF-N XION Nasopharingoskope, Germany). Each patient was placed in a seated or semi-seated position. As a first step, a bolus of semi-liquid consistency (jam or pureed fruit) was administered and then, secondly, a bolus with a liquid consistency (milk or juices). The patient was asked to hold the bolus in the oral cavity and to swallow it upon the command of the operator. The operation was repeated for the subsequent bolus. Each patient was given a score according to the Penetration Aspiration Scale (PAS) [18]. PAS is an 8-point scale, routinely performed in clinics and used to characterize depth and response to airway invasion during swallowing. The cut-off required to establish OD diagnosis was PAS ≥ 3.

### Statistical methods

The intra-rater reliability of DSA® and the inter-rater agreement of the evaluations of characteristics of swallowing dysfunction were determined by calculating Cohen’s kappa value. Sensitivity and specificity of DSA® relative to FEES for OD incidence were calculated in the target populations. Bayes’ theorem was used to calculate the positive predictive value (PPV), negative predictive value (NPV), positive likelihood ratio (LR +), negative likelihood ratio (LR-), pre-test probabilities (PrTP), and post-test probabilities (PoTP) of the DSA® test. The range of confidence interval (CI) was 95%. Protocol study was conducted according to the principles and rules laid down in the Declaration of Helsinki and its subsequent amendments.

## Results

All healthy volunteers (mean age 30 ± 7.5, 10 males and 10 females) were grade 0 at DSA® and presented with a safe and efficacious swallow at FEES. The study group consisted of 72 patients (mean age 72.2 ± 15.3, 35 males and 37 females). The Cohen’s kappa value for inter-rater reliability (two independent observers) for FEES and for intra-rater reliability of DSA® (one SLP) was, respectively, 0.91 and 0.97. With regard to the clinical bedside examination [19], the assessment of orofacial praxis abilities was within normality in 53 cases (74%) and with a deficit or not assessable in 19 cases (26%); tongue protrusion and strength were within normality in 62 cases (86%) and with deficit or not assessable in 10 cases (14%); voluntary cough was within normality in 48 cases (67%) and with deficit or not assessable in 24 cases (33%).

**Fig. 3:**
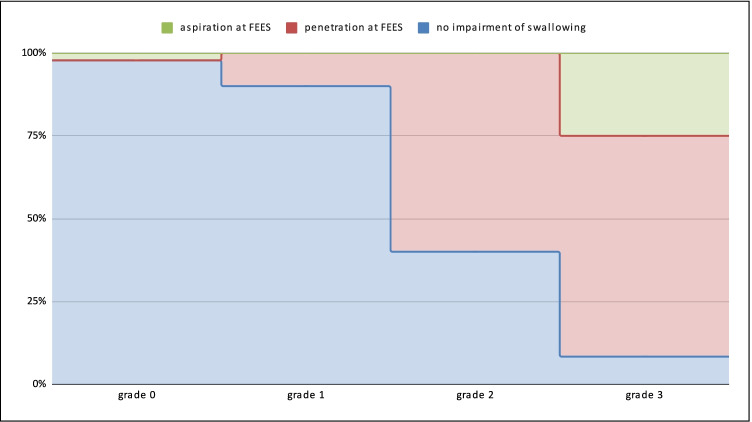
DSA—Dysphagia Standard Assessment and FEES outcome

FEES revealed 2/72 cases of hemi-laryngeal hypomobility, 9/72 cases of absent or deficient laryngeal reflex, and 11/72 cases of slow and difficult oral swallowing. For 62 patients the index test was administered from grade 0 and for 10 patients from grade 3. The outcomes of the index tests relative to the reference test results are reported in Table 2 and Fig. 3.

**Table 2:** Outcomes of the Dysphagia Standard Assessment DSA® according to FEES findings

FEES	*N*	Cough (%)	Wet voice (%)	≥ 5% Desaturation (%)
Healthy subjects	56	19.6	0	0
Aspiration	4	75	25	50
Penetration	12	87.5	31.2	25

Within the population of 72 patients with suspected OD, only 16 (22%) were actually affected by OD, of whom 11 (69%) had a high risk of aspiration/penetration and post-swallowing residue and 5 (31%) had a lower risk of aspiration/penetration but only moderate post-swallowing residue. Desaturation monitoring was useful in all those cases (33%) that had voluntary cough deficit and uncertain aspiration/penetration during screening test administration. A high percentage of the false positive results (11/12) were found to have been in patients with a slight impairment of swallowing (PAS 2) who were incorrectly identified by the test as positive.

Overall, the DSA® test showed an accuracy of 82%, a sensitivity of 0.93 (95% C.I. 0.84–0.97), and a specificity of 0.78 (95% C.I. 0.67–0.87). The PPV was 0.55 (95% C.I. 0.43–0.67); NPV was 0.97 (95% C.I. 0.90–0.99), LR + was 4.37 (95% C.I. 3.6–5.2); LR − was 0.08 (95% C.I. 0.06–0.09). PrTP of OD was 0.22 (95% C.I. 0.03–0.43), instead positive PoTP was 0.55 (95% C.I. 0.39–0.70) and negative PoTP was 0.02 (95% C.I. 0.17–0.27). The overall results of the main subgroups of patients are reported in Table 3.

**Table 3:** Dysphagia Standard Assessment DSA® outcomes

	*All sample*			*Cerebrovascular disease*			*Neurodegenerative diseases*		
		*Lower limit*	*Upper limit*		*Lower limit*	*Upper limit*		*Lower limit*	*Upper limit*
*Sensitivity*	0.938	0.848	0.978	0.833	0.611	0.952	1.000	0.828	1.000
*Specificity*	0.786	0.670	0.870	0.789	0.557	0.931	0.75	0.529	0.894
*P pre-test*	0.222	0.034	0.437	0.24	− 0.05	0.592	0.333	0.044	0.66
*P post-test* +	0.556	0.397	0.707	0.556	0.29	0.797	0.667	0.409	0.866
*P post-test − *	0.022	− 0.179	0.271	0.063	− 0.236	0.47	0.000	0.000	0.435
*Positive predictive value*	0.550	0.434	0.671	0.556	0.349	0.746	0.667	0.447	0.836
*Negative predictive value*	0.978	0.902	0.997	0.938	0.747	0.992	1.000	0.828	1.000
*Positive likelihood ratio*	4.375			3.950			4		
*Negative likelihood ratio*	0.08			0.211			0		

## Discussion

Currently, the ESPEN guidelines [6] indicate a timely evaluation of patients with neurological disorders for OD, preferably with FEES or VFSS, considering these, based on the current literature, as valid and safe methods for diagnosis. Timely evaluation allows practitioners to identify patients most at risk of aspiration and therefore are able to modify the viscosity of their food according to the European Society for Swallowing Disorder (ESSD) range. Increasing viscosity also aids the reduction in the risk of airway invasion and is a valid management strategy for OD [20]. In addition, it is necessary firstly to enhance the use of existing bedside screening tests used to evaluate swallowing function at the oropharynx and esophagus levels and secondly for bedside FEES evaluations to be undertaken by organized teams of adequately trained professionals required to meet the huge demand for dysphagia evaluation and treatment [21, 22].

### Primary outcome: reliability and validity of DSA® test

Regarding FEES, only about 22% of the patients examined were affected by OD. These data showed that the request for endoscopic evaluation of swallowing is very high compared to the number of cases that would actually need an examination. Instead, these resources could be used in targeted cases if BST was routinely performed in hospital wards. However, the systematic use of a test requires good reliability, consistent replication, and ease of administration. The DSA® test showed a 93% likelihood of identifying patients with dysphagia and a 78% likelihood of identifying healthy patients in a population that is neither homogeneous in terms of age nor in their pathology. Consideration should also be given with regard to PPV, since the prevalence of dysphagia is different between the groups examined. The predictive value is affected by this condition and, in fact, if a disease is considered pronounced within a population, the level of predictability for the same test, with equal sensitivity and specificity, grows when compared to a population where the frequency is lower. Faced with a non-homogeneous sample, the predictive values suggest that the probability of a patient turning out to be grade 0 and being healthy is 97%. Conversely, the probability of a grade of 1, 2, or 3 shows that dysphagia is less likely, at 55%, which explains why these patients should always be examined with FEES. These results lead us to think that the outcomes could be different if alternatively, we decided to apply the BST to only one type of target population. The likelihood ratios show the same trend; it is more likely that the patient who tests negative will be non-dysphagic than that of a patient who tests positive and has an undiagnosed swallowing disorder. The post-test probability of dysphagia with a positive test is, as stated, 55%; the post-test probability of non-dysphagia with a negative test is close to zero.

The DSA® test showed good accuracy (82%) in the whole sample, although it appears to have a lower validity in neurodegenerative diseases than in cerebrovascular diseases. In fact, the prevalence of any disease in the population can influence the predictive capacity of the test, which is why in neurodegenerative diseases where the prevalence of the disease (dysphagia) is higher [21], we see better predictive values.

### DSA® test and V-VST

The volume-viscosity swallowing test (V-VST) was designed in 2005 at Mataró Hospital, and several studies have evaluated the outcomes of the V-VST test in screening and diagnosing OD among different patients [12, 14, 16, 23, 24]. From recent evidence, V-VST had a diagnostic sensitivity for OD of 93.17%, specificity of 81.39%, and inter-rater reliability using Cohen’s kappa value of 0.77. The odds ratios for OD were LR + 5.01 and LR − 0.08 and the diagnostic odds ratio for OD was 51.18 [23]. According to the results in the literature, the DSA® test had a sensitivity of 93% and a specificity of 78%, with LR + 4.37 and LR − 0.08, although FEES was used as a reference test instead of VFSS. Relatively low values ​​of specificity and LR + indicate a high false positive rate and a low PPV value when the prevalence of OD is low. For this reason, the test was a good tool to exclude OD, but not to confirm the diagnosis.

### Oxygen desaturation analysis

Several studies have investigated oxygen saturation as a standalone tool or in combination with BST to determine the presence of a risk of OD and aspiration, but there are conflicting data on its usefulness [11, 25, 26]. Although the use of oxygen saturation could allow better analysis even of those patients with voluntary hypovalid or deficient cough and/or deficient laryngeal reflex, many other factors can influence the measurement of oxygen saturation [27]. Our results showed that only 50% of patients with aspiration and 25% of patients with penetration had oxygen desaturation > 5% at the test (Table 2). Although, as also found in the study by Clavè et al. [12], both oximetry and speech voice after swallowing did increase the diagnostic sensitivity of the test and the probability of identifying patients with silent aspirations or bolus penetration, however, the test was not effective in detecting a patient false negative with aspiration.

### Secondary outcome: analysis of test failure

Overall, the tests showed negative outcomes in 13/72 (18%) of patients (1 false negative and 12 false positive). Analyzing individual patients, the possible reasons for false negative test results can be traced back to the onset of raclage during the administration of the test in 7/12 cases, to lack of cooperation from the patient in 4/12 cases, and in one of the twelve cases to a patient with tracheostomy who needed SLP rehabilitation for swallowing after non-commotional head trauma. The false negative patient could be traced back to the absence of laryngeal sensitivity and cough reflex, and it was actually difficult to measure saturation as the patient was not cooperating. In all patients with no cough reflex, the presence of wet voice or oxygen values was supportive, but it is not clear whether one or more of these conditions were missing as test reliability varies. In fact, O2 saturation alone does not have sufficient diagnostic accuracy to support routine use, which is why silent aspirations may be missed by the test [28].

### Strengths and limitations of the DSA® test

One of the strengths of the DSA® test is the opportunity of not having to follow a fixed administration scheme but to proceed based on the patient’s risk of dysphagia and being able to choose whether to start with the administration of a thickened food (grade 3) or just water (grade 0). Starting from grade 0 is suitable for all patients who may have difficulty swallowing, while starting at grade 3 is appropriate for all patients who already have swallowing disorders. In addition, the ability to monitor oxygen saturation allows the test to be used even on patients at high risk of dysphagia, despite the limitations previously described.

The DSA® test also has its limitations: firstly, the duration of test administration, especially when starting from grade 0. Although choosing different starting degrees of liquid strength can reduce execution times, these are still quite long, on average 15–20 min, both for the preparation of the boluses and for their administration. A further limitation is the inability to positively test for all of the diseases we have investigated.

### Future directions: purpose of a flowchart for patient eligibility

**Fig. 4:**
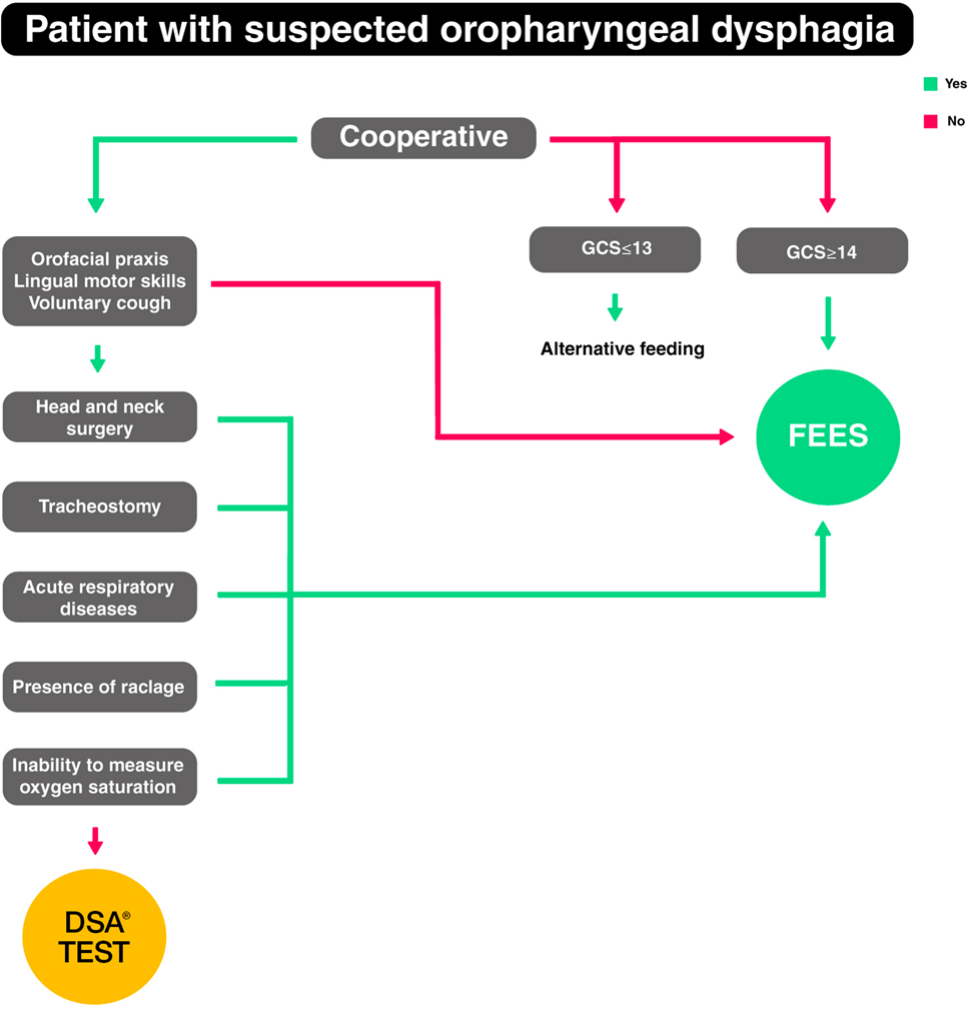
Flowchart for improving indications for DSA use

Overall, this study is a starting point that has allowed us to highlight false positives and negatives, and improved indications to apply the test safely, while excluding those patients who cannot exempt themselves from an instrumental evaluation. Based on our result analysis and direct patient observations, a flowchart has been developed to improve indications for DSA use (see Fig. 4). More in depth patient collaboration is the first point: if the patient is not cooperative, FEES should be considered rather than a DSA® test. If the non-cooperation is caused by GCS < 14, an alternative feeding method, e.g., nasogastric tube or parenteral nutrition should instead be immediately considered. If the patient cooperates, orofacial praxis, lingual motor skills, and voluntary cough should be evaluated; FEES should be preferred if deficient; DSA® test if valid. On the contrary, it is always recommended to perform FEES in patients undergoing head and neck surgery, in neurological patients with pathologies characterized by involuntary movement disorders (e.g., dyskinesias in PD, Huntington’s disease, dystonia), in patients with tracheostomy or with acute respiratory disease, in patients with gastroesophageal reflux disease raclage or smoker’s raclage, and in patients with problems of desaturation or with absence of cough reflex. In other cases, the DSA® test is recommended. However, if the test gives a grade 1–2–3 result, FEES would always be recommended to identify any silent aspirations (Fig. 4). Further studies are needed to understand whether, if applying this flowchart, test failure rates could be lower or if further patient selection evaluations are needed for the safe use of volume viscosity tests as a screening method for OD.

## Conclusions

According to our preliminary results, the DSA® test has shown good outcomes in discriminating for the presence or absence of OD with a wide spectrum of applicability in the sample of patients examined, with some limitations that may be overcome by the choice of a target population. In fact, analyzing the outcomes, some cases had negative results for the same factors. Considering the increased demand for a broad and repeatable applicability of a bedside screening test for OD, the authors have provided a flowchart to address patient eligibility.
